# Adaptation of the carbamoyl-phosphate synthetase enzyme in an extremophile fish

**DOI:** 10.1098/rsos.201200

**Published:** 2020-10-14

**Authors:** Lewis J. White, Gemma Sutton, Asilatu Shechonge, Julia J. Day, Kanchon K. Dasmahapatra, Mary E. Pownall

**Affiliations:** 1 Biology Department, University of York, York YO10 5DD, UK; 2 Tanzania Fisheries Research Institute, PO BOX 98, Kyela, Mbeya, Tanzania; 3 Department of Genetics, Evolution and Environment, University College London, Darwin Building, Gower Street, London WC1E 6BT, UK

**Keywords:** urea, carbamoyl-phosphate synthetase, *Alcolapia*, extremophile, fish

## Abstract

Tetrapods and fish have adapted distinct carbamoyl-phosphate synthase (CPS) enzymes to initiate the ornithine urea cycle during the detoxification of nitrogenous wastes. We report evidence that in the ureotelic subgenus of extremophile fish *Oreochromis Alcolapia*, CPS III has undergone convergent evolution and adapted its substrate affinity to ammonia, which is typical of terrestrial vertebrate CPS I. Unusually, unlike in other vertebrates, the expression of CPS III in *Alcolapia* is localized to the skeletal muscle and is activated in the myogenic lineage during early embryonic development with expression remaining in mature fish. We propose that adaptation in *Alcolapia* included both convergent evolution of CPS function to that of terrestrial vertebrates, as well as changes in development mechanisms redirecting *CPS III* gene expression to the skeletal muscle.

## 1. Introduction

In living organisms, protein metabolism results in the production of nitrogenous wastes which need to be excreted. Most teleosts are ammonotelic, excreting their toxic nitrogenous waste as ammonia across gill tissue by diffusion. As an adaptation to living on land, amphibians and mammals are ureotelic, using liver and kidney tissues to convert waste ammonia into the less toxic and more water-soluble urea, which is then excreted in urine. Other terrestrial animals such as insects, birds and reptiles are uricolotic and convert nitrogenous waste into uric acid, which is eliminated as a paste; a process which requires more energy but wastes less water [1].

While most adult fish are ammonotelic, the larval stages of some teleosts excrete nitrogenous waste as both ammonia and urea before their gills are fully developed [2]. Additionally, some adult fish species such as the gulf toad fish (*Opsanus beta*; [3]) and the African catfish (*Clarias gariepinus*; [4]) also excrete a proportion of their nitrogenous waste as urea. This is usually in response to changes in aquatic conditions, such as high alkalinity. It has been shown experimentally that high external pH prevents diffusion of ammonia across gill tissue [5,6]. Unusually, the cichlid fish species in the subgenus *Alcolapia* (described by some authors as a genus but shown to nest within the genus *Oreochromis*) [7], which inhabit the highly alkaline soda lakes of Natron (Tanzania) and Magadi (Kenya), are reported to be 100% ureotelic [8,9].

Once part of a single palaeolake, Orolonga [10], Lakes Natron and Magadi are one of the most extreme environments supporting fish life, with water temperatures up to 42.8°C, pH approximately 10.5, fluctuating dissolved oxygen levels, and salt concentrations above 20 parts per thousand [11]. *Alcolapia* is the only group of fish to survive in these lakes, forming a recent adaptive radiation including the four species: *Alcolapia grahami* (Lake Magadi) and *A. latilabris*, *A. ndlalani* and *A. alcalica* (Lake Natron) [11,12]. The harsh environment of the soda lakes presents certain physiological challenges that *Alcolapia* have evolved to overcome, including the basic need to excrete nitrogenous waste. While other species are able to excrete urea in response to extreme conditions, none do so to the level of *Alcolapia* [13,14], and unlike facultative ureotelic species, the adaptation of urea production and excretion in *Alcolapia* is considered fixed [15]. Moreover, the heightened metabolic rate in *Alcolapia*, a by-product from living in such an extreme environment [8,16], requires an efficient method of detoxification.

*Alcolapia* and ureotelic tetrapods (including humans) detoxify ammonia using the ornithine urea cycle (OUC) where the mitochondrial enzyme carbamoyl-phosphate synthetase (CPS) is essential for the first and rate-limiting step of urea production [17]. This enzyme, together with the accessory enzyme glutamine synthase, provide an important switch regulating the balance between ammonia removal for detoxification and maintaining a source of ammonia for the biosynthesis of amino acids [18]. CPS has evolved into two biochemically distinct proteins: in terrestrial vertebrates CPS I uses ammonia as its preferential nitrogen donor, while in teleosts CPS III accepts glutamine to produce urea during larval stages (reviewed Zimmer *et al.* [2]). While CPS I/III are mitochondrial enzymes and part of the urea cycle, CPS II is present in the cytosol catalysing the synthesis of carbamoyl phosphate for pyrimidine nucleotide biosynthesis. *CPS I/III* are syntenic, representing orthologous genes; their somewhat confusing nomenclature is based on the distinct biochemical properties of their proteins. *CPS I/III* genes from different vertebrate species clade together, separate from *CPS II* (electronic supplementary material, figure). For simplicity, we will continue to refer to fish, glutamine binding CPS as CPS III and tetrapod, ammonia binding CPS as CPS I. The teleost CPS III binds glutamine in the glutamine amidotransferase (GAT) domain using two amino acid residues [19], subsequently, the nitrogen source provided by the amide group is catalysed by a conserved catalytic triad; Cys-His-Glu [20]. In terrestrial vertebrates CPS I lacks a complete catalytic triad and can only generate carbamoyl-phosphate in the presence of free ammonia [21]. This change in function from glutamine binding CPS III to ammonia binding CPS I is believed to have evolved in the stem lineage of living tetrapods, first appearing in ancestral amphibians [21].

In tetrapods and most fish, the OUC enzymes are largely localized to the liver [22], the main urogenic organ [23]. *Alcolapia* are different, and the primary site for urea production in these extremophile fishes is the skeletal muscle [24]. Notably, glutamine synthase activity is reportedly absent in *Alcolapia* muscle tissue. The kinetic properties of CPS III in *Alcolapia*, therefore, differ from that of other teleosts in that it preferentially uses ammonia as its primary substrate, having maximal enzymatic rates above that of binding glutamine (although it is still capable of doing so) as opposed to in other species where the use of ammonia yields enzymatic rates of around 10% to that of glutamine [24]. These rates are similar to ureotelic terrestrial species, where CPS I preferentially binds ammonia and is incapable of using glutamine [20].

Here, we report the amino acid sequence of two *Alcolapia* species (*A. alcalica* and *A. grahami*) that reveals a change in CPS III substrate binding site. In addition, we show that the expression of *Alcolapia* *CPS III* in skeletal muscle arises early in embryonic development where transcripts are restricted to the somites, the source of skeletal muscle in all vertebrates, and migrating myogenic precursors. We discuss changes to the structure of functional domains and modular gene enhancers that probably underpin evolutionary changes in *Alcolapia* CPS III substrate binding and the redirection of gene expression from the hepatogenic to myogenic lineage [25]. Our findings point to adaptation in *Alcolapia* including both convergent evolution of CPS function to that of terrestrial vertebrates, as well as changes in development mechanisms redirecting *CPS III* gene expression to the skeletal muscle.

## 2. Methods

### 2.1. Experimental animals

Fieldwork at Lake Natron, Tanzania, was conducted during June and July of 2017 to collect live specimens of the three endemic species in an attempt to produce stable breeding populations of these fishes in the UK. Live fish were all collected from a single spring (site 5 [11,26]) containing all three species found in Lake Natron and identified using morphology as described in Seegers & Tichy [12]. A stand-alone, recirculating aquarium was adapted to house male and female *A. alcalica* in 10 or 30 l tanks at a constant temperature of 30°C, pH 9 and salt concentration of 3800 µS at the University of York.

### 2.2. Expression of *CPS III* in adult tissues

Reverse transcription–polymerase chain reaction (RT-PCR) was used to determine the presence of *CPS III* in different tissues (gill, muscle, liver, brain) of three different adult *A. alcalica*. RNA was extracted from dissected tissues with TriReagent (Sigma-Aldrich) to the manufacturers' guidelines. For cDNA synthesis, 1 µg of total RNA was reverse transcribed with random hexamers (Thermo Scientific) and superscript IV (Invitrogen). PCR was performed on 2 µl of the above cDNA with Promega PCR master mix and 0.5 mM of each primer (forward: CAGTGGGAGGTCAGATTGC, reverse: CTCACAGCGAAGCACAGGG). Gel electrophoresis of the PCR products determined the presence or absence of *CPS III* RNA.

### 2.3. *In situ* hybridization

For the production of antisense probes, complementary to the mRNA of *CPS III* to use in *in situ* hybridization, the above 399 bp PCR product was ligated into PGem-tEasy and transformed into the *Escherichia coli* strain DH5*α*. This was linearized and *in vitro* run-off transcription was used to incorporate a DIG-labelled UTP analogue. To determine the temporal expression of these proteins in *A. alcalica*, embryos were collected at different stages of development (2, 4 and 7 days post fertilization (between 15 and 20 for each stage)), fixed for 1 hour in MEMFA (0.1 M MOPS pH 7.4, 2 mM EGTA, 1 mM MgSO_4_, 3.7% formaldehyde) at room temperature and stored at −20°C in 100% methanol. For *in situ* hybridization, embryos were rehydrated and treated with 10 µg mg^−1^ proteinase *K* at room temperature. After post-fixation and a 2 h pre-hybridization, embryos were hybridized with the probe at 68°C in hybridization buffer (50% formamide (Ambion), 1 mg ml^−1^ total yeast RNA, 5×SSC, 100 µg ml^−1^ heparin, 1× denharts, 0.1% Tween-20, 0.1% CHAPS, 10 mM EDTA. Embryos were extensively washed at 68°C in 2×SSC + 0.1% Tween-20, 0.2×SSC + 0.1% Tween-20 and maleic acid buffer (MAB; 100 mM maleic acid, 150 mM NaCl, 0.1% Tween-20, pH 7.8). This was replaced with pre-incubation buffer (4× MAB, 10% BMB, 20% heat-treated lamb serum) for 2 h. Embryos were incubated overnight (rolling at 4°C) with fresh pre-incubation buffer and 1/2000 dilution of anti-DIG coupled with alkaline phosphatase (AP) (Roche). These were then visualized by the application of BM purple until staining had occurred.

### 2.4. Sequence analysis of CPS III

cDNA was produced from the RNA extracted from whole embryos using the above method for *A. alcalica* and *A. grahami*. Multiple primer pairs (electronic supplementary material, table S1) were used to amplify fragments of *CPS III* from the cDNA via PCR and the products sent for sequencing. The coding region of *CPS III* was then constructed using multiple alignments against the *CPS I* and *III* from other species. The amino acid sequence was then examined for potential changes which could predict the functional differences seen in *Alcolapia*. Phylogenetic analysis was also used to confirm the *Alcolapia* genes analysed here are *CPS III* (electronic supplementary material, figure S1). To determine potential changes in the promoter region, a 3500 bp section of genome (accession number NCBI: MW014910) upstream of the transcriptional site start of *CPS* from *A. alcalica* (unpublished genome), *Oreochromis niloticus* (Nile tilapia), *Xenopus tropicalis* (western clawed frog) and *Danio rerio* (zebrafish) genomes was accessed on Ensembl, aligned and examined for binding sites specific to the muscle transcription factor Myod1 (E-boxes) which preferentially binds paired E-boxes in the enhancer regions of myogenic genes with the consensus motif CAG(G/C)TG, as well as E-boxes more broadly (CANNTG). The published genomes of *O. niloticus*, *D. rerio* and *X. tropicalis* were accessed using Ensembl, whereas the *Alcolapia* genome was constructed from whole-genome sequences.

## 3. Results

### 3.1. *CPS III* expression is activated early in the skeletal muscle lineage in *A. alcalica*

**Figure 1.**
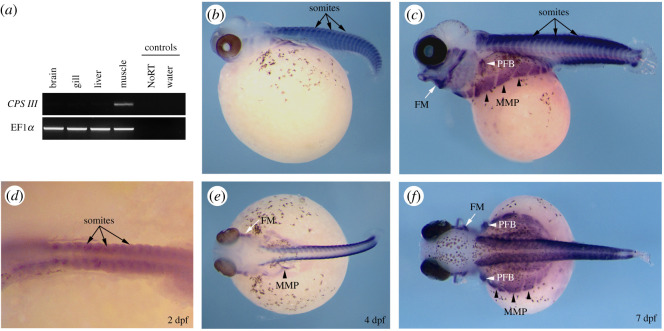
Expression analysis of carbamoyl-phosphate synthetase III (*CPS III*) from adult tissues and developing embryos of *Alcolapia alcalica.* (*a*) Reverse transcriptase PCR and gel electrophoresis showing the muscle-specific expression of *CPS III*, EF1*α* shown as normalization control. (*b*–*f*) Lateral (*c* and *e*) and dorsal (*b*, *d* and *f*) views of *in situ* hybridization for *CPS III* in developing *A. alcalica* embryos at different stages (number of days post fertilization [dpf] indicated). The blue colour indicates the detection of mRNA. The black/brown is endogenous pigment apparent in the retina and the chromatophores. Black arrows show somites, black arrowheads indicate region of migrating muscle progenitors (MMP), white arrows show facial muscle (FM) and white arrowheads indicate developing pectoral fin bud (PFB). Black dots around the yolk and on the body are chromatophores (pigment cells).

Analysis of gene expression of *CPS III* in dissected tissues of three adult *A. alcalica* shows that transcripts were only detected in adult muscle (figure 1*a*). *In situ* hybridization methods on *A. alcalica* embryos at different stages were carried out to investigate whether this restricted muscle expression was established during development (figure 1*b–f*). Blue coloration indicates hybridization of the complementary RNA probe and shows the strongest expression in the developing somites along the body axis (black arrows). Expression was also detected in migratory muscle precursors (MMP; black arrowheads), which go on to form the body wall and limb musculature, and in the developing pectoral fin buds (white arrows). All regions of the embryo that show expression of *CPS III* are in the muscle lineage indicating that in *A. alcalica* *CPS III* expression is restricted to muscle tissues in both adults and the developing embryo.

**Figure 2.**
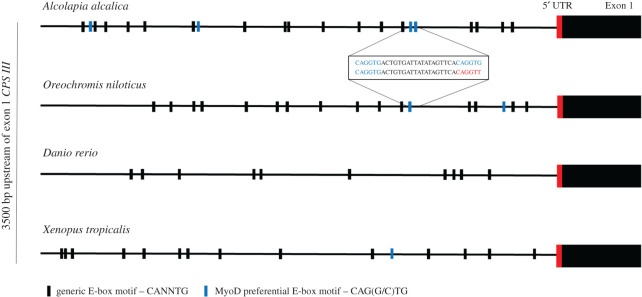
Sequence comparison of a 3500 bp region upstream of exon 1 of *CPS III*. Presence of generic E-box sites with the consensus motif of CANNTG are annotated by black bars and MyoD preferential E-box motifs of CAG(G/C)TG are represented by blue bars. Insert shows sequence of *Alcolapia alcalica* potential paired E-box, MyoD enhancer (blue nucleotides) compared to *Oreochromis niloticus* which has a single MyoD E-box upstream of a ‘presumptive' MyoD E-box (red nucleotides).

Many muscle-specific genes are activated during development by the muscle-specific transcription factor, MyoD. The promoter region of *CPS III* (3.5 kb upstream of the transcriptional start site) in *A. alcalica* was compared to that in *O. niloticus*, *X. tropicalis* and *D. rerio* (figure 2). Examination of this region revealed a putative paired E-box MyoD binding site 940–970 bases (CAGGTGACTGTGATTATATAGTTCACAGGTG) upstream of the transcriptional start site of *CPS III* only in *Alcolapia* species. Intriguingly, while no pair of MyoD E-boxes were found in the upstream region of any other species examined, *O. niloticus* does have a single MyoD E-box motif in the same region upstream of *CPS III*, and within 19 bases of this is a CAGGTT motif which a single point mutation would convert into a pair of E-boxes (CAGGTGACTGTGATTATATAGTTCACAGGTT). This suggests that it is possible that MyoD could bind and activate transcription of *CPS III* in the muscle of *Alcolapia* species, but not in the closely related *O. niloticus*.

### 3.2. Convergent evolution in the adaptive function of CPS III

**Figure 3.**
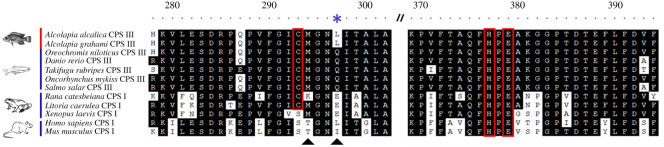
Multiple amino acid alignment of residues 278–397 (aligned to *Alcolapia alcalica*) of carbamoyl-phosphate synthetase I and III from a number of tetrapod and teleost species, respectively. Conserved amino acids are shaded in black, amino acids in the catalytic triad are boxed in red and arrowheads indicate residues vital for glutamine utilization of the catalytic triad. The blue asterisk indicates the divergent glutamine binding residue in *Alcolapia* species that probably results in a functional change (inability to bind glutamine).

Sequence analysis of *A. alcalica* and *A. grahami* CPS III revealed a discrepancy in the catalytic triad compared to the published sequence for CPS III in *A. grahami* (accession number NCBI: AF119250). The coding region for *A. alcalica* and *A. grahami* was cloned and sequenced (accession numbers NCBI: MT119353, MT119354). Our data confirmed the error in the published sequence of *A. grahami* CPS III and shows *Alcolapia* species maintain a catalytic triad essential for catalysing the breakdown of glutamine (red boxes in figure 3). However, similar to terrestrial vertebrate CPS I which lack either one but usually both residues essential for binding glutamine for utilization by the catalytic triad (arrowheads in figure 3), *Alcolapia* also lack one of these residues (asterisk in figure 3). This amino acid sequence is consistent with a change in function permitting *Alcolapia* CPS III to bind and catalyse ammonia directly, an activity usually restricted to terrestrial vertebrate CPS I, as elucidated by extensive previous biochemical analyses [20,21].

## 4. Discussion

While most teleosts are ammonotelic, larval fish can convert ammonia to urea for excretion and to do so express the genes coding for the enzymes of the OUC, including CPS III [27]. Later these genes are silenced in most fish. In the rare cases where urea is produced in adult fish, the OUC enzymes are expressed in the liver [23]; however, there are some reports of expression in non-hepatic tissues [28,29]. We report here the expression of *CPS III* in the muscle of adult *A. alcalica*, which is consistent with the detection of CPS III protein and enzyme activity in muscle of *A. grahami* [24]. We also find conserved changes to the amino acid sequence which explains the convergent evolution of *A. alcalica* and *A. grahami* CPS III function with CPS I in terrestrial vertebrates. This conserved change in both *Alcolapia* species suggests that the adaptations in the OUC are likely to have evolved in the ancestral species inhabiting palaeolake Orolongo during the period of changing aquatic conditions (over the past 10 000 years) that led to the extreme conditions currently found in Lakes Natron and Magadi.

### 4.1. Activation of *CPS III* in the myogenic lineage

We find that the expression of *CPS III* is activated in somites and in migratory muscle precursors that will form body wall and limb musculature (indeed expression is seen in developing limb buds). All skeletal muscle in the vertebrate body is derived from the somites, and these *CPS III* expression patterns are similar to those of muscle-specific genes like myosin, actins and troponins [30–32].

Muscle-specific expression of *CPS III* in *A. alcalica* embryos is a remarkable finding as most ureotelic species convert nitrogenous waste to urea in the liver [8,20]. The expression of *CPS III*, the first enzyme in the OUC, in muscle tissue is probably significant for supporting the high catabolism in a fish species with the highest recorded metabolic rate [33]. There are few reports of some OUC gene expression or enzyme activity in non-hepatic tissue including muscle [28,29,34]; nonetheless, other fish species only evoke the activity of the OUC when exposed to high external pH or during larval stages [13,14,35,36], and even then, urea production is never to the high level of activity occurring in *Alcolapia* [24]. There is some heterogeneity of the expression patterns of *CPS III* during the development of different species in the teleost lineage; for example, *D. rerio* has reported expression in the body [37], *Oncorhynchus mykiss* (rainbow trout) shows expression in the developing body but not in hepatic tissue [38] and *C. gariepinus* (African catfish) had *CPS III* expression detected in the dissected muscle from larvae [4]. The early and sustained expression of *CPS III* in the muscle lineage is at this point an observation unique to *Alcolapia*.

Skeletal muscle-specific gene expression is activated in cells of the myogenic lineage by a family of bHLH transcription factors, including MyoD [30]. MyoD binds specifically at paired E-boxes in the enhancers of myogenic genes with a preference for the consensus motif of CAG(G/C)TG [39,40]. MyoD is known to require the cooperative binding at two E-boxes in close proximity, to modulate transcription of myogenic genes [41]. The presence of a pair of E-boxes in *Alcolapia*, upstream of a gene which has switched to muscle-specific expression, is suggestive that MyoD is driving expression early in development. Enhancer modularity is a known mechanism for selectable variation [42] and although a single MyoD binding site does not define an enhancer, MyoD is known to interact with pioneer factors and histone deacetylases to open chromatin and activate gene transcription in the muscle lineage [40,43]. Experimental analysis to determine the activity of any regulatory sequences upstream of OUC genes in different species would shed light on the significance of putative transcription factor binding sites. This approach could also address another intriguing question as to the elements that drive the post-larval silencing of OUC genes in most fish species [37], an area with only minimal research, especially when compared to the well-characterized promoter region in mammalian species, for instance, Christoffels *et al.* [44]. A further instance of an extremophile organism redirecting the expression of a hepatic enzyme to muscle tissue occurs in the crucian carp [45]. Under conditions of anoxia, this species switches to anaerobic metabolism, producing ethanol as the end product of glycolysis [46,47]. This is associated with the expression of *alcohol dehydrogenase* in muscle [48]. Together with our findings, this potentially reveals an example of convergent evolution whereby the muscle becomes the site for detoxifying by-products of metabolism. Elucidating any mechanisms that may include modular enhancers that facilitate the adaptation of gene regulation in response to changing environmental conditions will be of significant interest.

### 4.2. Convergent evolution of adaptive CPS III function

CPS proteins catalyse the production of carbamoyl-phosphate as the first step in nitrogen detoxification by accepting either glutamine or ammonia as a nitrogen donor [17]. Teleost CPS III binds glutamine: the nitrogen source provided by the amide group of glutamine is catalysed by the conserved catalytic triad Cys-His-Glu in the glutamine amidotransferase (GAT) domain in the amino terminal part of CPS [20]. In terrestrial vertebrates, CPS I lacks the catalytic cysteine residue and only generates carbamoyl-phosphate in the presence of free ammonia [21]. Although CPS in *Alcolapia* shares the most sequence identity with fish CPS III (figure 3), its ammonia binding activity is more similar in function to terrestrial vertebrate CPS I [20,24]. This adaptation to preferentially bind ammonia over glutamine supports efficient waste management in a fish with an exceptionally high metabolic rate [33]. CPS I in terrestrial vertebrates have amino acid changes in the catalytic triad which explains their binding ammonia over glutamine; a reduction in glutamine binding capacity drives the use of ammonia [21]. Here, we show that *Alcolapia* maintain the catalytic triad, but (similar to mouse and human) lack one of the two residues required for efficient glutamine binding, weakening its affinity to glutamine and driving the use of ammonia as a primary substrate.

The interesting observation that bullfrog (*Rana catesbeiana*) CPS I retains the catalytic triad, but lacks the two additional conserved amino acids required for glutamine binding, has led to the suggestion that the change from preferential glutamine to ammonia binding originally evolved in the early tetrapod lineage [21]. A further frog species, the tree frog *Litoria caerulea*, retains its catalytic triad and only one of the two residues required for glutamine binding has been altered, weakening its affinity for glutamine and allowing for direct catabolism of ammonia [49]. Much the same as in *Alcolapia*, *L. caerulea* CPS I is still capable of using glutamine to some extent which lends further support to the notion that the evolutionary transition from CPS III to CPS I occurring in amphibians and the early tetrapod lineage. The changes in the protein sequence of *Alcolapia* CPS III represents a convergent evolution in this extremophile fish species, with acquired changes in functionally important domains which probably also evolved in early terrestrial vertebrate CPS I.

## 5. Conclusion

*Alcolapia* have acquired multiple adaptations that allow continued excretion of nitrogenous waste in a high pH environment. Among these is the novel expression of *CPS* in skeletal muscle, as well as the acquisition of mutations that change its function. Sequence evidence indicates that like terrestrial vertebrates, and unique among fish, *Alcolapia* CPS III is capable of binding and catalysing the breakdown of ammonia to carbamoyl-phosphate; a convergent evolution of *CPS* function. The mechanism by which the novel and unique expression of CPS in muscle evolved is probably a function of enhancer regions of *A. alcalica* and *A. grahami* that result in its regulation by muscle regulatory factors to direct CPS expression in the myogenic lineage during embryonic development. Environmentally driven adaptations have resulted in changes in both the expression and activity of CPS III in *Alcolapia* that underpin its ability to turn over nitrogenous waste in a challenging environment while maintaining a high metabolic rate.

## Supplementary Material

Table of primers for sequencing CPS III

Reviewer comments

## Supplementary Material

Phylogenetic analysis of CPSI, II and III

## Supplementary Material

Sequences of CPS in A. grahami and A. alcalica (CDS)

## Data accessibility

Sequence data has been made available on NCBI (accession numbers: MT119353, MT119354) and in the electronic supplementary material, files.
