# Supplementary material for: Adaptation of the carbamoyl-phosphate synthetase enzyme in an extremophile fish
Source: R Soc Open Sci. 2020 Oct 14;7(10):201200. doi: 10.1098/rsos.201200 (PMC7657897; doi:10.1098/rsos.201200)
Supplement: Table of primers for sequencing CPS III [file rsos201200supp1.docx]

Supplementary table 1: Primer pairs used to amplify fragments of *CPS* *III* by PCR from the embryonic RNA extracted from *A. alcalica* and *A. grahami.* These were subsequently sequenced and aligned using BIOEDIT.

| Segment of gene amplified (bases) | Forward primer | Reverse primer |
| --- | --- | --- |
| 1 (1-871) | ATGGCAAAAATCCTCCAAGCTG | CAAATACTGGCTGGGGACGATC |
| 2 (782-1644) | CTAGTGGACCAGGAGATCCATC | CTGCCTGTCCTCTGTTGCC |
| 3 (1567-2436) | CGTGGCATCTTGGACCAGTAC | GGCCTTCTGCATACTCTCC |
| 4 (2344-3255) | CACGGCATGTCTCATGAAA | GTCGATCTGCAGGGGGCTCG |
| 5 (3167-3987) | CAGTGGGAGGTCAGATTGC | CTCACAGCGAAGCACAGGG |
| 6 (3830-4521) | TCAACGTGGCAACCAAAGTG | CTAACCCTGCTGGCTAGACCC |
