# Supplementary material for: Adaptation of the carbamoyl-phosphate synthetase enzyme in an extremophile fish
Source: R Soc Open Sci. 2020 Oct 14;7(10):201200. doi: 10.1098/rsos.201200 (PMC7657897; doi:10.1098/rsos.201200)
Supplement: Phylogenetic analysis of CPSI, II and III [file rsos201200supp2.docx]

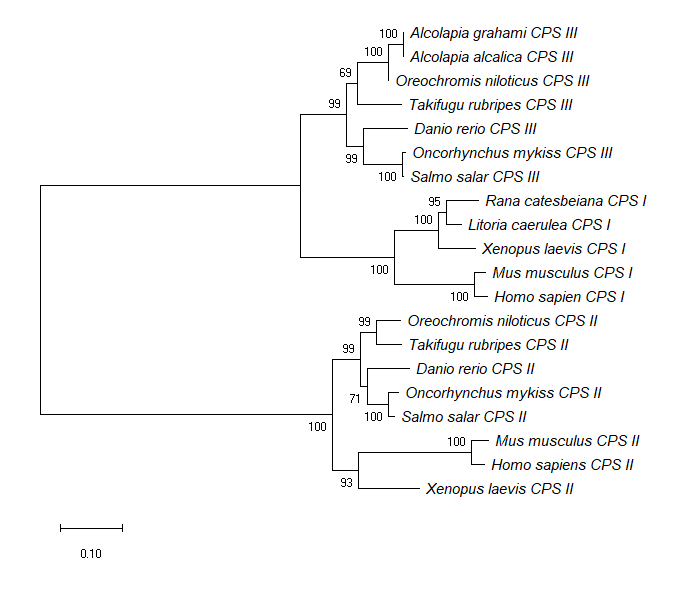


**Supplementary Figure 1:** Phylogenetic analysis of the amino acid sequences of CPS proteins from a range of fish and tetrapod species. Analysis was conducted on MEGAX by the maximum likelihood method and the LG model with gamma distribution. All positions containing gaps were excluded and the tree was verified with 100 bootstrap replicates (shown on branches). CPS I/III produce a single clade separate from the CPS II amino acid sequences from a number of the same species. Both *Alcolapia* CPS III, which were sequenced in this study, clade as expected with CPS III from other fish species.
